# Vitamin D and Sjögren’s Disease: Revealing the Connections—A Systematic Review and Meta-Analysis

**DOI:** 10.3390/nu15030497

**Published:** 2023-01-18

**Authors:** Mislav Radić, Ela Kolak, Hana Đogaš, Andrea Gelemanović, Dora Bučan Nenadić, Marijana Vučković, Josipa Radić

**Affiliations:** 1 Internal Medicine Department, Rheumatology, Allergology, and Clinical Immunology Division, University Hospital of Split, 21000 Split, Croatia; 2 Department of Internal Medicine, School of Medicine, University of Split, 21000 Split, Croatia; 3 Nutrition and Dietetics Department, University Hospital of Split, 21000 Split, Croatia; 4 Internal Medicine Department, Nephrology and Haemodialysis Division, University Hospital of Split, 21000 Split, Croatia; 5 Mediterranean Institute for Life Sciences (MedILS), 21000 Split, Croatia

**Keywords:** Sjögren’s disease, Sjögren’s syndrome, vitamin D, cholecalciferol, systematic review, meta-analysis

## Abstract

Background: The aim of the present review was to summarize the current evidence about the impact of vitamin D deficiency on pathology and clinical manifestations of Sjögren’s disease (SD). Methods: Databases PubMed, Web of Science, Scopus, and Cochrane library were searched for studies assessing the levels of vitamin D in SD patients using the following keywords: (vitamin D OR calciferol OR cholecalciferol OR 25-hydroxyvitamin D OR 25-hydroxycholecalciferol OR calcidiol OR calcitriol OR 1,25-dihydroxycholecalciferol) AND (Sjögren’s Syndrome OR Sjögren’s disease) accessed on 20 September 2022. Out of 248 retrieved studies, following the systematic review methodology and defined inclusion and exclusion criteria, 9 clinical studies were eligible to be included in the present review: 4 of them case-control, 4 cross-sectional, and 1 cohort study. Results: Nine studies totaling 670 SD patients and 857 healthy controls were eligible for meta-analysis with moderate to high methodological quality as determined by the Newcastle–Ottawa Quality Scale (NOS). According to the obtained results, a high prevalence of hypovitaminosis D was observed in SD patients when compared to healthy controls (95% CI −10.43, −2.39; *p* < 0.01). Conclusion: Available evidence points to lower levels of vitamin D in patients with SD in comparison to healthy controls. However, further studies are necessary to understand the underlying mechanisms associated with the role of vitamin D in the development and disease severity of SD.

## 1. Introduction

Vitamin D_3_ or cholecalciferol in its natural form enters the body through dietary intake (20%) or is synthesized by the skin (80%) upon UVB exposure from 7-dihydrocholesterol to pre-vitamin D_3_ and activated by the hydroxylation process. The first hydroxylation occurs in the liver, where pre-vitamin D_3_ metabolizes to 25-hydroxyvitamin D_3_ (25(OH)D_3_) which is the main circulating form of vitamin D and one of the most reliable biomarkers of vitamin D status. 25(OH)D_3_ is then transported by vitamin D binding protein to the kidney where it is hydroxylated to fully active metabolite 1,25-dihydroxyvitamin D_3_ (1,25(OH)_2_D_3_) responsible for most of the vitamin D effects [1,2].

To better understand the effects of vitamin D, two different modes of action are distinguished—calcemic and non-calcemic actions. Regarding calcemic actions, vitamin D increases serum calcium concentration by inducing proteins involved in active intestinal absorption and phosphate absorption, maintains normal blood calcium concentration by mobilizing calcium from bone (requires the presence of parathyroid hormone (PTH)), and induces calcium reabsorption in the distal renal tubule (also requires the presence of the PTH). [3]. The non-calcemic effects have not yet been explored but can be speculated via the expression of vitamin D receptors (VDRs), which mediate the genomic actions of vitamin D [4]. VDRs have been found in parathyroid cells, pituitary cells, promyelocytes, lymphocytes, keratinocytes, ovarian, and colon cells, while evidence for their presence in skeletal muscle, cardiac muscle, and liver was inconclusive [3]. Many tissues, among them the colon, the pancreatic islets, the breasts, prostate, vascular smooth muscle cells, macrophages, and malignant and immune cells, also possess 1-α-hydroxylase and can produce 1,25(OH)_2_D_3_ locally. This has suggested that 1,25(OH)_2_D_3_ has a role in modulating innate and adaptive immunity and affecting endocrine systems [4].

**Figure 1:**
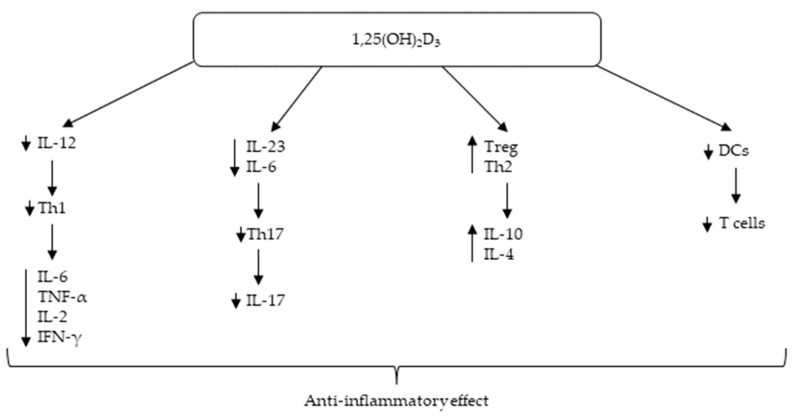
Immunomodulatory effect of vitamin D. Abbreviations: 1,25(OH)_2_D_3_—1,25-dihydroxyvitamin D_3_, DCs—dendritic cells, IFN-γ—interferon γ, IL-2—interleukin, IL-4—interleukin 4, IL-6—interleukin 6, IL-10—interleukin 10, IL-12—interleukin 12, IL-17—interleukin 17, IL-23—interleukin 23, TNF-α—tumor necrosis factor α, Th1—T helper cell 1, Th2—T helper cell 2, Th17—T helper cell 17, Treg—T regulatory cell.

In recent years, the immunomodulatory role of vitamin D has become a topic of interest, especially in the context of autoimmune diseases. Innate and adaptive immune responses make up the immune system. Activation of toll-like receptors in polymorphonuclear cells, macrophages, monocytes, and epithelial cells is considered the innate immune response. This activation leads to the induction of cathelicidin, which is in epithelial and myeloid cells and is also induced by 1,25(OH)_2_D_3_, thus promoting the innate response [5]. The effect of vitamin D on adaptive immunity is much broader. The main component of adaptive immunity is the activation of T and B lymphocytes [6]. Dysregulation of T-cell response and chronically activated T cells results in immune-mediated diseases. Therefore, 1,25(OH)_2_D_3_ has been found to be an important immune response regulator, mainly mediating T-cell responses [7]. In general, 1,25(OH)_2_D_3_ reduces the maturation of dendritic cells and therefore decreases their ability to activate T-cells [5]. 1,25(OH)_2_D_3_ also has a suppressor role in adaptive immunity by downregulating the synthesis of cytokines [8]. Indirectly, it diminishes the response and development of Th1 lymphocytes by suppressing IL-12, and as a result, it suppresses the production of IL-2, IL-6, TNF-α, and IFN-γ. It also favors the anti-inflammatory cytokine IL-10 by upregulating T regulatory cells (Treg) and Th2 lymphocyte activity [2,8]. 1,25(OH)_2_D_3_ downregulates the development of Th17 lymphocytes by suppressing IL-23 and IL-6 production [5]. Excessive numbers or increased activation of Th17 or Th1 lymphocytes have been linked to a variety of autoimmune diseases such as type I diabetes mellitus, multiple sclerosis, Graves’ disease, systemic lupus erythematosus (SLE), rheumatoid arthritis (RA) and connective tissue disease [6,9]. The effects of vitamin D on T cells are all summarized in Figure 1.

All these observations indicate the importance of vitamin D levels in the treatment and outcomes of patients with autoimmune diseases. The connection between vitamin D deficiency and supplementation with immune-mediated diseases has long been studied. Several studies have found that patients with autoimmune diseases have lower serum levels of 25(OH)D_3_ than their healthy counterparts [2]. However, the causal relationship is not yet clear; it is unclear whether low serum levels of 25(OH)D_3_ pose a risk of developing an autoimmune disease or whether these low serum levels are the consequence of the disease [10].

Sjögren’s disease (SD) is a systemic autoimmune disease in which most patients present primarily with ocular dryness and xerostomia (dry mouth) due to focal lymphocytic infiltration of the exocrine glands [11]. SD is an often underestimated and common disease, affecting approximately 0.1–0.6% of the adult population with a female-to-male ratio of 9:1 [12]. One of the more serious aspects of primary Sjögren’s disease (pSD) is a 10- to 50-fold increased risk of developing lymphoma, i.e., 2–9% of patients with pSD develop lymphoma. Some studies even suggest that low vitamin D levels are associated with the development of lymphoma and peripheral neuropathy [13]. However, when specifically studying current research on the connections between vitamin D and SD, the results are inconclusive. For example, in 2018, Li et al. conducted a meta-analysis of studies that compared vitamin D levels in SD patients and a control group. The meta-analysis had several limitations, among them a small number of included studies (30-year search span), and the results could not be pooled by adjusting for confounders (age, ethnicity, season, outdoor activity, diet intake, etc.) because not all studies generated adjusted values [14]. Given the paucity of relevant studies on this topic and the low quality of the evidence, there is a need to further investigate the associations between vitamin D and SD. Therefore, the aim of the present study was to assess and evaluate the current studies on vitamin D levels in SD and, consequently, to stimulate further research in this field.

## 2. Materials and Methods

### 2.1. Search Strategy

The present review was conducted following the requirements of the Preferred Reporting Items for Systematic Reviews and Meta-Analysis (PRISMA) Statement [15]. The authors (E.K. and H.Đ.) independently performed a literature search based on the PICO components (population; intervention; comparison; outcome). A literature review was performed on 20 September 2022 searching for studies assessing the serum levels of vitamin D in SD. PubMed, Web of Science, Scopus, and Cochrane library databases were searched using the following keywords: (vitamin D OR calciferol OR cholecalciferol OR 25-hydroxyvitamin D OR 25-hydroxycholecalciferol OR calcidiol OR calcitriol OR 1,25-dihydroxycholecalciferol) AND (Sjögren’s Syndrome OR Sjögren’s disease). No filters or limitations were used. Furthermore, the reference list of the primary studies and associated reviews was manually searched to identify additional relevant publications.

### 2.2. Literature Selection

The following criteria had to be met for inclusion:

- study design: prospective or retrospective cohort study, randomized controlled trial, or observational studies;
- studies with a control group of either healthy or sicca syndrome subjects;
- studies assessing serum 25(OH)D_3_ levels in SD patients and controls;
- an available full version of the paper online;
- papers available in the English language.
- Therefore, the exclusion criteria were:
- abstracts and conference abstracts, letters, comments, case reports, reviews, or meta-analyses;
- studies not exploring patients with SD;
- studies with unavailable serum 25(OH)D_3_ levels for patients or controls;
- patients or healthy controls taking vitamin D supplements;
- studies with a control group consisting of patients with various comorbidities;
- unavailable serum 25(OH)D_3_ levels for patients or controls;
- papers not available online in full version or not available in the English language.

### 2.3. Data Extraction

From the included studies, the following data were extracted by two separate authors (E.K. and H.Đ.): the first author’s surname and the year of publication; the basic characteristics such as study design, sample size, mean age, and mean disease duration; and the levels of serum 25(OH)D_3_ for patients and controls.

### 2.4. Outcome Measures

The primary outcome of the evaluation was the serum levels of 25(OH)D_3_ in patients with SD. In analyzing the primary outcome in studies fulfilling the inclusion criteria, further evaluation was conducted to assess any secondary outcomes among the parameters analyzed in selected studies pertaining to the role of vitamin D in SD.

### 2.5. Quality Assessment

To assess the quality of the included studies, the Newcastle–Ottawa Scale (NOS) was used [16,17]. The NOS evaluated three categories—selection, comparability, and exposure/outcome—with a maximum overall score of 9 or 10 points for each study, depending on the study design. Each study design (cohort, case-control, and cross-sectional) had an adjusted form that evaluated these three categories. A score of 7 or higher indicates a high quality, a score of 4 to 6 indicates a moderate risk of bias, and a score of 0 to 3 indicates a high risk of bias. Two reviewers (H.Đ. and E.K.) conducted the evaluation independently, reaching a final assessment with differences resolved by consensus.

### 2.6. Statistical Analysis

Meta-analysis was performed in the free software environment for statistical computing R version 4.0.0 [18] using package meta v6.0-0 [19]. To evaluate the vitamin D levels in patients with SD in comparison to controls, a random-effects meta-analysis model was applied with inverse variance weighting, and the mean difference (MD) with a 95% confidence interval (CI) was obtained. Mean differences were considered significant if the *p*-value < 0.05 in the test for overall effect. Heterogeneity between studies was evaluated using the *I*^2^ index, and if the test for heterogeneity was significant (*p*-value < 0.05) it meant that there was significant heterogeneity between the studies. To evaluate the potential sources of heterogeneity between the studies, subgroup analyses were performed based on the geographical location where the studies were performed (Europe, Turkey, East Asia). In addition, a subset of studies that used the data for women only was analyzed separately. Forest plots were used to depict the results of meta-analyses.

## 3. Results

### 3.1. Literature Search Result

Two hundred and forty-eight records were retrieved after an extensive literature search, among them 68 from PubMed, 9 from Cochrane library, 71 from Web of Science, and 100 from Scopus. After 40 duplicates were removed, the titles and abstracts of the remaining studies were examined, and a total of 149 studies were discarded based on the study design. Furthermore, after careful and independent full-text assessment by the two authors (E.K. and H.Đ.), 53 studies were excluded according to the already established inclusion and exclusion criteria, with potential discrepancies resolved by discussion until a consensus was reached. Moreover, 3 additional studies were identified from the references of the relevant studies.

**Figure 2:**
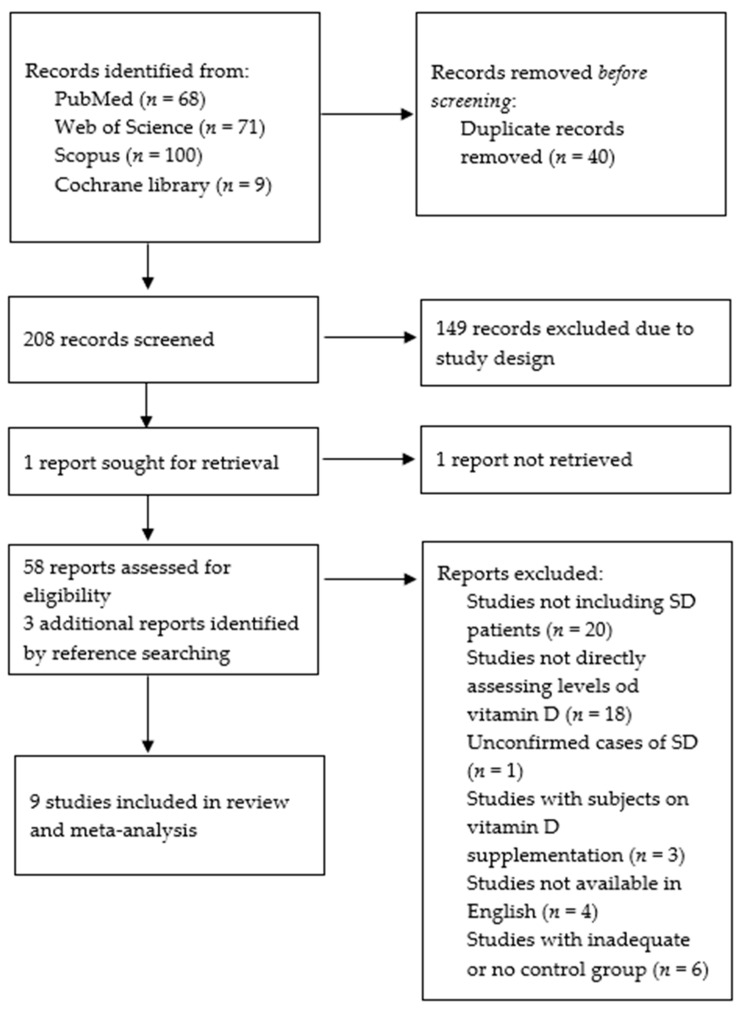
Structural outline of the study selection and exclusion process. Abbreviations: SD—Sjögren’s disease, *n*—number.

Finally, 9 articles were selected, as shown in Figure 2, and reviewed by the senior researcher (M.R.).

### 3.2. Characteristics of the Studies

Nine studies totaling 670 SD patients and 857 healthy controls were included in the present meta-analysis. Among them, 4 were case-control, 4 were cross-sectional, and 1 was a cohort study. Regarding geographical location, 5 studies were conducted in Europe, 2 in Turkey, and 2 in East Asia. A summary of the selected studies is shown in Table 1.

### 3.3. Risk of Bias and Quality Assessment

Of the 9 included studies, 2 studies scored 6, 4 studies scored 7, 2 studies scored 9, and 1 study scored 10, as indicated in Table 1. Overall, all the included studies had moderate-to-high methodological quality.

### 3.4. Meta-Analysis of the Differences in Serum 25(OH)D_3_ Levels between SD Patients and Healthy Controls

**Figure 3:**
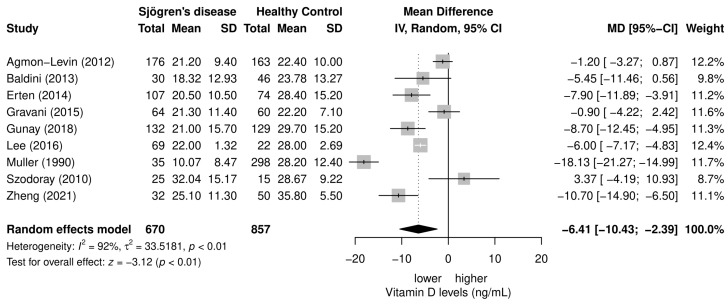
Meta-analysis of vitamin D levels in patients with SD in comparison to controls [18,19,20,21,22,23,24,25,26].

All included studies reporting the vitamin D levels in patients with SD compared to healthy controls could enter the meta-analysis, and the results of the random-effects meta-analysis are shown in Figure 3. Vitamin D levels were lower in patients with SD compared to healthy controls, 21.28 ± 4.26 ng/mL vs. 27.46 ± 4.38 ng/mL, respectively (mean difference = −6.41; 95% CI −10.43, −2.39; *p* < 0.01). Heterogeneity between studies was shown to be substantial (*I*^2^ = 92%; *p* < 0.01).

**Figure 4:**
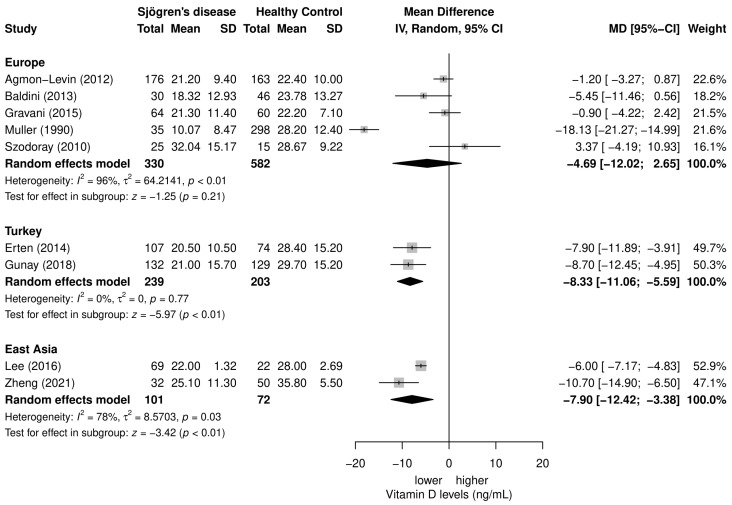
Subgroups meta-analysis based on geographical location of included studies evaluating the vitamin D levels in SD patients in comparison to controls [18,19,20,21,22,23,24,25,26].

Due to the large heterogeneity between studies, a subgroup meta-analysis was performed based on the geographical location of the included studies to evaluate the potential source of the observed heterogeneity. Five studies conducted their research on the European population, two studies on the Turkish population, and two studies on the East Asian population. The results of the geographical subgroup meta-analysis are shown in Figure 4. Overall, only subgroups of the Turkish and East Asian populations showed a significant overall effect of lower vitamin D levels in patients with SD in comparison to healthy controls (MD = −8.33; 95% CI −11.06, −5.59; *p* < 0.01 and MD = −7.90; 95% CI −12.42, −3.38; *p* < 0.01; respectively), and interestingly, only a subgroup of studies performed on the Turkish population showed no heterogeneity. Studies on the European population showed substantial heterogeneity (*I*^2^ = 96%, *p* < 0.01) and did not show a significant overall effect (*p* = 0.21).

**Figure 5:**
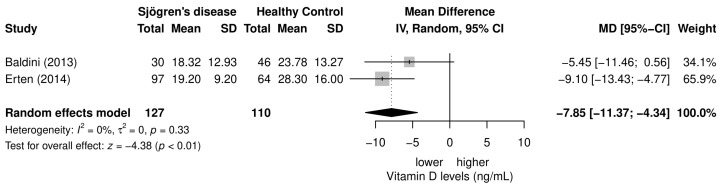
Subset meta-analysis of vitamin D levels in female patients with SD in comparison to female controls [21,22].

Two studies reported the data for women only and these two studies were used in the subset analysis. Results of the female subset meta-analysis are shown in Figure 5, and these results correspond with the full sample size (vitamin D levels in female patients with SD were 18.76 ± 2.63 ng/mL vs. vitamin D levels in female healthy controls were 26.04 ± 1.93 ng/mL; mean difference = −7.85; 95% CI −11.37, −4.34; *p* < 0.01), but there was no observed heterogeneity between these studies (*I*^2^ = 0%, *p* = 0.33).

## 4. Discussion

In recent years, the immunomodulatory role of vitamin D has become a topic of particular interest, especially in the context of autoimmune diseases. A relationship between suboptimal levels of vitamin D and several autoimmune diseases such as RA, SLE, and systemic sclerosis has been previously described, whereas the data about vitamin D deficiency and the overall role of vitamin D in the clinical manifestations of SD are scarce and often contradictory. Therefore, the aim of this review and meta-analysis was to explore the current knowledge about vitamin D levels in SD and, consequently, to stimulate further research in this field.

The results of the present meta-analysis, based on a statistical analysis of the results of nine studies, show that vitamin D levels are generally lower in SD patients compared with healthy controls, but with a significant overall heterogeneity, indicating several confounding factors. Similar results were observed in the meta-analysis by Kuo et al. [29]. On the other hand, Li et al. previously reported comparable serum vitamin D levels between SD patients and controls [14]. The reason for the resulting differences could be the number of included studies and subjects, as well as the inclusion and exclusion criteria applied since the previously mentioned meta-analyses included subjects supplemented with vitamin D and subjects with another autoimmune disease in addition to SD.

Possible confounding factors in the context of SD and vitamin D levels could be gender, age, ethnicity, sun exposure, disease activity and disease duration, blood pressure, and smoking, but considering the provided data from included studies only ethnicity and female gender entered subgroups analysis.

We hypothesized that subjects’ geographical background and ethnicity could be related to serum vitamin D levels in SD. According to the results of the meta-analysis of subgroups based on geographical background, the Turkish and East Asian subgroups had significantly lower vitamin D levels, while the European subgroup showed no significance in vitamin D levels when compared to healthy controls. However, only the Turkish subgroup did not show high heterogeneity.

Vitamin D status is strongly influenced by race and geographic location, among other factors. Geographic locations closer to the equator have higher and longer overall sunlight exposure, implying that vitamin D synthesis is higher because of longer as well as higher UV exposure [30]. Due to the high melatonin concentration, darker-pigmented skin requires longer UV exposure to produce the same amount of vitamin D compared to lighter-pigmented skin with a lower melatonin concentration [30]. Therefore, darker-skinned people living farther from the equator are at higher risk for vitamin D deficiency, as noticed in previous research, and these factors should be considered [31]. Given the somewhat conflicting results in the subgroup meta-analysis, the importance of considering confounding factors is highlighted and implores further research. Even though strong female propensity is characteristic of SD with a female-to-male ratio varying from 9:1 to 20:1 across the literature [32], only two studies reported data for women, which correspond with the full sample size but without significant heterogeneity observed [23,24]. Interestingly, Erten et al. reported significantly lower vitamin D levels in female-affected patients when compared to the female controls, with the same association not observed in the males [24]. This difference may not be related to sex but to body composition, considering that adipose tissue may represent a depot for vitamin D and thus reduce its bioavailability [33]. Data coming from the National Health and Nutrition Examination Survey (NHANES) 2005 to 2006 conducted with 8306 US adults with available vitamin D measurements suggested that the prevalence of vitamin D deficiency was higher (53.8%) in subjects with obesity when compared to those with normal body weight (33%) [34]. According to the recent prospective cohort study, which included 250 females and 250 males matched by age, serum vitamin D levels were significantly lower in females compared to males in all body mass index (BMI) classes. Furthermore, females with vitamin D deficiency had a higher fat mass percentage in comparison to males with vitamin D deficiency however, vitamin D concentrations were inversely correlated with a fat-mass percentage in both sexes [35].

Two of the nine included studies observed a negative association between vitamin D levels and disease activity as measured by the Eular Sjögren’s syndrome disease activity index (ESSDAI) [26,28]. Although the number of mentioned studies is insufficient for further statistical analysis, the observed results point to the new possible role of vitamin D, which especially gains significance since the reduction in disease activity in RA and SLE patients with a significant reduction in anti-dsDNA positivity and a biomarker of clinical flares was achieved by vitamin D supplementation [36].

Although not the primary focus of the present review, it is important to mention the findings of individual studies in the context of the relationship between vitamin D and the clinical manifestations of SD. Lymphocytic infiltration of exocrine glands is characteristic of SD, but also numerous clinical manifestations varying from mild symptoms such as sicca symptoms of dry eyes and dry mouth to severe ones involving multiple organ systems are associated with this chronic disease [32]. Several studies included in the present review noticed a correlation between serum vitamin D levels and extraglandular involvement, mainly expressed through the nervous system [21,26,28]. Zheng et al. observed inverse correlation between vitamin D levels and peripheral nervous system domain with the occurrence of peripheral nervous system involvement between 10% and 46% in patients affected by pSD [28]. On the other hand, Agmon-Levin et al. determined a correlation between neuropathy and low levels of vitamin D [22]. Reported rates of peripheral neuropathy in patients with SD generally range between 1.6 and 31% [37]. Vitamin D has shown neuroprotective and neuroactive properties, and, as such, is highly active in regulating cell differentiation, proliferation, and peroxidation in the brain [38]. Furthermore, the high local concentrations of vitamin D could increase nerve growth factor in Schwann cells, glial cells that generate myelin sheath and provide trophic support to the neurons, through VDR [39]. Taken together, all the data suggest a possible role for vitamin D in the peripheral neuropathy of SD and incite further exploration.

Another finding of the study by Agmon-Levin et al. was a connection between lymphoma and low vitamin D levels in pSD patients, who are at a 9- to 16-fold increased risk for non-Hodgkin lymphoma [22]. Suboptimal levels of serum vitamin D were associated with an increased risk for non-Hodgkin lymphoma in the general population as well [40]. Moreover, pretreatment vitamin D deficiency was associated with adverse clinical outcomes in these patients [41]. Vitamin D and its analogues showed an antiproliferative effect on lymphoma cell lines and diminished the expression of the VDR [42]. According to the results of the International Lymphoma Epidemiology Consortium meta-analysis, sun exposure could protect against the development of non-Hodgkin lymphoma and its B- or T-cell subtypes due to the intensified vitamin D metabolism caused by UV light exposure. The same effect could not be reached when dietary vitamin D was measured [43]. Surely, dietary intake of vitamin D is not considered a reliable predictor of vitamin D status since it is affected by the efficiency of cutaneous biosynthesis [44]. In addition, SD patients are prone to gastrointestinal symptoms similar to those seen in inflammatory bowel disease due to food hypersensitivity, especially to wheat and dairy products [45]. Thus, the adequacy of dietary vitamin D intake raises a question. These results highlight the unsuspected roles of vitamin D in the clinical manifestations of the disease and point to possible areas for future research.

The present review has several advantages, most notably the observation of serum vitamin D levels in SD patients and healthy controls by ethnicity, and despite the large heterogeneity, the included studies had moderate-to-high methodological quality. The present review has some limitations. Considering the application of strict inclusion and exclusion criteria to achieve high-quality review, the number of eligible studies for statistical analysis was limited. In addition, serum vitamin D levels may be influenced by gender, age, sun exposure, body composition, blood pressure, and smoking factors that have not been considered in the overall evidence base associated with SD. Although the present study demonstrated an association between the presence of SD and suboptimal serum vitamin D levels, causality was not established. In addition, it is important to mention publication bias. Studies with statistically significant results are more likely to be published than studies without significant results, even though they might have clinical significance. In addition, the use of databases for literature searches could result in sampling bias due to data mismatches with respect to the hypothesis as well as data incompleteness.

## 5. Conclusions

In conclusion, the present study showed that individuals affected by SD have lower serum vitamin D levels compared with healthy controls. In observing the ethnicity and geographical background of the subjects, no correlation was found to serum vitamin D levels in SD. However, due to relatively reduced diversity and number of applicable studies, no definite conclusion could be made regarding ethnical and geographical background influence. Further studies are necessary to elucidate the actual effect of all the confounding factors that affect vitamin D levels in SD (ethnicity, gender, age, body composition, sun exposure, etc.) in order to have an individualized approach to each patient regarding supplementation and treatment. Supplementation of this vitamin could be beneficial in alleviating the symptoms associated with SD and should be considered, particularly in the specific subgroups of SD such as patients with multiple comorbidities. Given the overall paucity of available studies observing the role of vitamin D in SD and multiple organ involvement associated with SD, further research in the form of randomized controlled trials is necessary to establish a role of vitamin D in the pathophysiology of SD.

## Figures and Tables

**Table 1:** Summary of the selected clinical studies.

Study	Country	Study Design	Sample Size (SD/HC)	Age (Years; SD/HC)	Disease Duration (Years)	NOS
Muller [20], 1990	Denmark	Cross-sectional	35/298	51/NA	NA	7/10
Szodoray [21], 2010	Hungary	Case-control	25/15	56.4	NA	7/9
Agmon-Levin [22], 2012	Several European countries	Case-control	176/163	NA	NA	7/9
Baldini [23], 2013	Italy	Prospective monocentric cross-sectional	30/46	NA	NA	9/10
Erten [24], 2014	Turkey	Case-control	107/74	45.24 ± 8/43.52 ± 8.89	44.1 ± 26.9	7/9
Gravani [25], 2015	Greece	Cohort study	64/60	57.2 ± 12.4/56.4 ± 7.8	8.4 ± 7.0	6/9
Lee [26], 2016	Korea	Case-control	69/22	NA	8.7 ± 0.78	6/9
Günay [27], 2018	Turkey	Analytical cross-sectional	132/129	52/51.6	NA	10/10
Zheng [28], 2021	China	Retrospective cross-sectional	32/50	43 ± 14/40 ± 12	3.3	9/10

Abbreviations: SD—Sjögren’s disease, HC—healthy controls, NOS—Newcastle–Ottawa Scale, NA—not applicable.

## Data Availability

Not applicable.
